# Pore shape and sorption behaviour in mesoporous ordered silica films^1^

**DOI:** 10.1107/S1600576716013698

**Published:** 2016-09-23

**Authors:** Gerhard Fritz-Popovski, Roland Morak, Parvin Sharifi, Heinz Amenitsch, Oskar Paris

**Affiliations:** a Institute of Physics, Montanuniversität Leoben, Franz-Josef-Strasse 18, 8700 Leoben, Austria; b Max-Planck-Institut für Kohlenforschung, Kaiser-Wilhelm-Platz 1, 45470 Mülheim an der Ruhr, Germany; c Institute for Inorganic Chemistry, Graz University of Technology, Stremayrgasse 9/V, A-8010 Graz, Austria

**Keywords:** sorption behaviour, pore shape, mesoporous silica films, grazing-incidence small-angle X-ray scattering, GISAXS, spin coating, dip coating

## Abstract

Mesoporous silica films templated by pluronic P123 were prepared using spin and dip coating. The ordered cylindrical structure within the films deforms due to shrinkage during calcination. Grazing-incidence small-angle X-ray scattering (GISAXS) measurements reveal that both the unit cell and the cross section of the pores decrease in size, mainly normal to the surface of the substrate, leading to elliptical cross sections of the pores with axis ratios of about 1:2. Water take-up by the pores upon changing the relative humidity can be monitored quantitatively by the shift in the critical angle of X-ray reflection as seen by the Yoneda peak.

## Abstract 2

The shape and sorption behaviour of pores in mesoporous ordered silica films are determined from grazing-incidence small-angle X-ray scattering data.

## 1. Introduction   

Nanoporous materials with well defined meso- and/or micropore sizes and shapes have been widely investigated for potential applications in catalysis separation, gas storage and electrical energy storage, as well as sensors and actuators. Probably the best control over nanostructural parameters within a wide range of pore sizes is reached with soft-templating methods, leading to ordered arrays of pores with remarkable control over pore shape and monodispersity (Zhao *et al.*, 2013 ▸). The characterization of such systems is mostly done with gas sorption methods which deliver sorption isotherms that can be interpreted in terms of porosity, specific surface area and pore-size distribution (Lowell *et al.*, 2004 ▸). However, in the case of thin porous films on substrates, standard gas sorption methods such as volumetric or gravimetric sorption of (mostly N_2_) gas are usually not applicable in a nondestructive way owing to the low amount of material available. Therefore, for thin films other methods are required to retrieve information on the pore-filling fraction as a function of relative gas pressure. Examples that have been successful in reaching this goal are ellipsometric porosimetry *via* the change in the index of refraction (Mogilnikov & Baklanov, 2002 ▸; Boissiere *et al.*, 2005 ▸), and X-ray reflectometry *via* the change in the critical angle of total reflection (Dourdain & Gibaud, 2005 ▸). Still, the models used to interpret sorption data are highly dependent on the details of the nanostructure of the pores, and therefore small-angle X-ray scattering (SAXS) supported by transmission electron microscopy (TEM) is usually employed to obtain more direct information on the pore structure (Grosso *et al.*, 2001 ▸; Falcaro *et al.*, 2004 ▸; Innocenzi *et al.*, 2005 ▸). Grosso *et al.* (2001 ▸) have shown that, for two-dimensional hexagonal mesoporous thin silica films, SAXS and grazing-incidence SAXS (GISAXS) corroborated by TEM are indeed very valuable methods to obtain detailed information on the pore structure. In particular, they have shown that the drying (Gibaud *et al.*, 2004 ▸) and calcination of the films lead to a change in the two-dimensional hexagonal symmetry of the pore lattice due to anisotropic shrinkage of the films. This must also be accompanied by a change in the pore shape, from cylinders with an originally circular cross section towards an elliptical cross section. The shape change due to anisotropic shrinkage was later analysed in more detail by Boissiere *et al.* (2005 ▸) for a cubic mesopore lattice. They used the macroscopic contraction of the film as measured by ellipsometry to draw indirect conclusions on the elliptical distortion of the originally spherical pores, and developed a model for the quantitative interpretation of sorption isotherms measured with ellipsometric porosimetry. Chavez Panduro *et al.* (2014 ▸) have shown that the GISAXS pattern of templated films can be better explained if one takes pore deformation into account. So far, however, no detailed direct analysis of pore shape changes due to shrinkage has been reported.

Here, we report the detailed analysis of a mesoporous silica film with an ordered two-dimensional hexagonal pore lattice by GISAXS. We show that, for cylindrical pores lying preferentially within the film plane, direct information on the anisotropic form factor of the pores can be obtained from the intensity of the Bragg reflections, and thus the shape of the pore cross section can be reconstructed independently of the change in the lattice parameters. Combining this independent information on pore size, shape and lattice parameters, we can easily calculate important parameters such as the specific surface area and pore volume fractions. Finally, we also demonstrate that the shift of the Yoneda peak (Yoneda, 1963 ▸) in the GISAXS patterns measured during a change in relative humidity can be used to reconstruct a sorption isotherm directly from the GISAXS data.

## 2. Experimental   

The sample preparation was based on evaporation-induced self-assembly approaches (Brinker *et al.*, 1999 ▸; Dourdain *et al.*, 2005 ▸; Yan *et al.*, 2007 ▸). A solution of 1.75 g of tetraethoxysilane in 1 g of ethanol and 1.25 g of water (acidified with HCl, pH 1.25) was stirred for 1 h. It was then mixed with a second solution of 580 mg of Pluronic P123 (Sigma–Aldrich) in 26.85 g of ethanol and stirred for another 2 h, after which 1.9 g of water acidified to pH 1.25 was added. Spin-coated films were prepared by spreading 30 µl of precursor solution on a silicon wafer substrate and rotating it for 30 s at 5000, 2000 or 500 r min^−1^, where lower rotation speeds lead to thicker films (Emslie *et al.*, 1958 ▸). Dip-coated films were obtained by immersing the substrate and withdrawing it at 2, 6, 8 or 10 mm s^−1^, which is in the Landau–Levich regime, where higher withdrawal rates lead to thicker films (Brinker *et al.*, 1991 ▸). All samples were calcined in air using a heating rate of 2 K min^−1^ up to 723 K and maintaining this temperature for 4 h.

Samples were degassed *in vacuo* for several hours before being transferred into a humidity chamber (Sharifi *et al.*, 2014 ▸), which was evacuated and flushed with dry air. The humidity was controlled by a mixture of air at 3 and 95% relative humidity at 298 K, which was changed at a rate of approximately 0.5% min^−1^.

GISAXS measurements were conducted at the Austrian SAXS beamline of the radiation source Elettra (Trieste, Italy) (Amenitsch *et al.*, 1998 ▸). The sample-to-detector distance was set to 1500 mm and the X-ray wavelength was 0.155 nm. A silver behenate (thickness ∼0.1 mm) reference sample (Huang *et al.*, 1993 ▸) was attached to the window of the humidity cell to monitor the stability of the experimental setup. Scattering patterns were measured with a two-dimensional pixel detector (Pilatus 1M, Dectris Ltd, Baden, Switzerland) for 20 s each, followed by a waiting time of 40 s. The incidence angle was approximately 0.4° and it was monitored for each measurement by the position of the specular reflection on the detector.

Cross sections of the films were prepared with focused ion beam cutting using an AURIGA Crossbeam Workstation (Zeiss). Scanning electron microscopy images of the sample cross sections were taken with the same instrument, with the electron microscope operated at a voltage of 2 keV, in order to determine the thicknesses of the films (Table 1 ▸). This was done at the identical positions where the GISAXS measurements had been performed previously.

## 3. Data evaluation   

**Figure 1:**
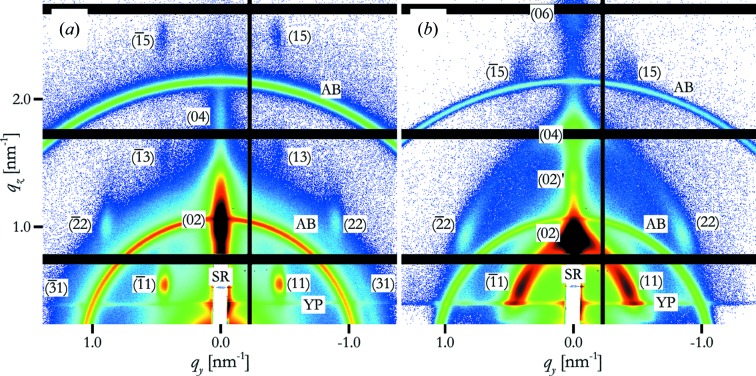
GISAXS patterns of (*a*) the spin-coated sample produced at 5000 r min^−1^ and (*b*) the dip-coated sample produced at a withdrawal speed of 10 mm s^−1^. Peaks are indicated by Miller indices of a rectangular lattice. The horizontal line labelled YP is the Yoneda peak and the rings labelled AB are from the silver behenate standard. SR is the specular reflection. The black grid is due to insensitive areas of the detector. The peak (02)′ indicates the (02) peak caused by the beam reflected from the sample surface.

The measured scattering patterns (Fig. 1 ▸) show peaks, rings and a horizontal line at the bottom. The beam-stop blocked most of the intense signal at low angles in the specular direction, but was sufficiently transparent to allow observation of the specular reflection, which was used to monitor the incidence angle. The rings are caused by the silver behenate standard being measured simultaneously with the sample and were used to calibrate the centre of the primary beam. The tilt of the substrate surface relative to the detector coordinate system around the axis defined by the primary beam was determined from the tilt of the Yoneda peak (horizontal line; Lazzari, 2009 ▸) on the detector image. The peaks at the detector are Bragg reflections (Bragg & Bragg, 1913 ▸) of the ordered pore lattice, which will be analysed below. They are arranged in a rectangular lattice and are indexed by the corresponding Miller indices.

### 3.1. Integrated peak intensity   

The peaks on the detector were approximated by a two-dimensional normal distribution:
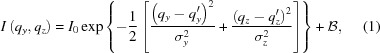
where *I*_0_ is the intensity at the maximum, 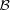 is a constant background, and σ_*y*_ and σ_*z*_ are its standard deviations in the horizontal and vertical directions, respectively. The position of the centre of each peak is given by the values 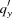 and 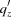. Note that the axes of the elliptical cross section of the peak are aligned with the axes of the coordinate system, *i.e.* any mis­alignment of the peaks is neglected.

In the case of the (02) peak, and if the (11) peaks overlapped with the Yoneda peak, the constant background used in equation (1) was replaced by one with a linear decay in the *q_z_* direction. This was necessary because sample roughness caused a decaying vertical streak which was still strong at the position of the (02) peak. The flank of the Yoneda peak could also cause a non-constant background in the vertical direction.

The positions 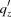 of the peaks were corrected for reflection and refraction effects based on the distorted-wave Born approximation (DWBA) (Tate *et al.*, 2006 ▸).

**Figure 2:**
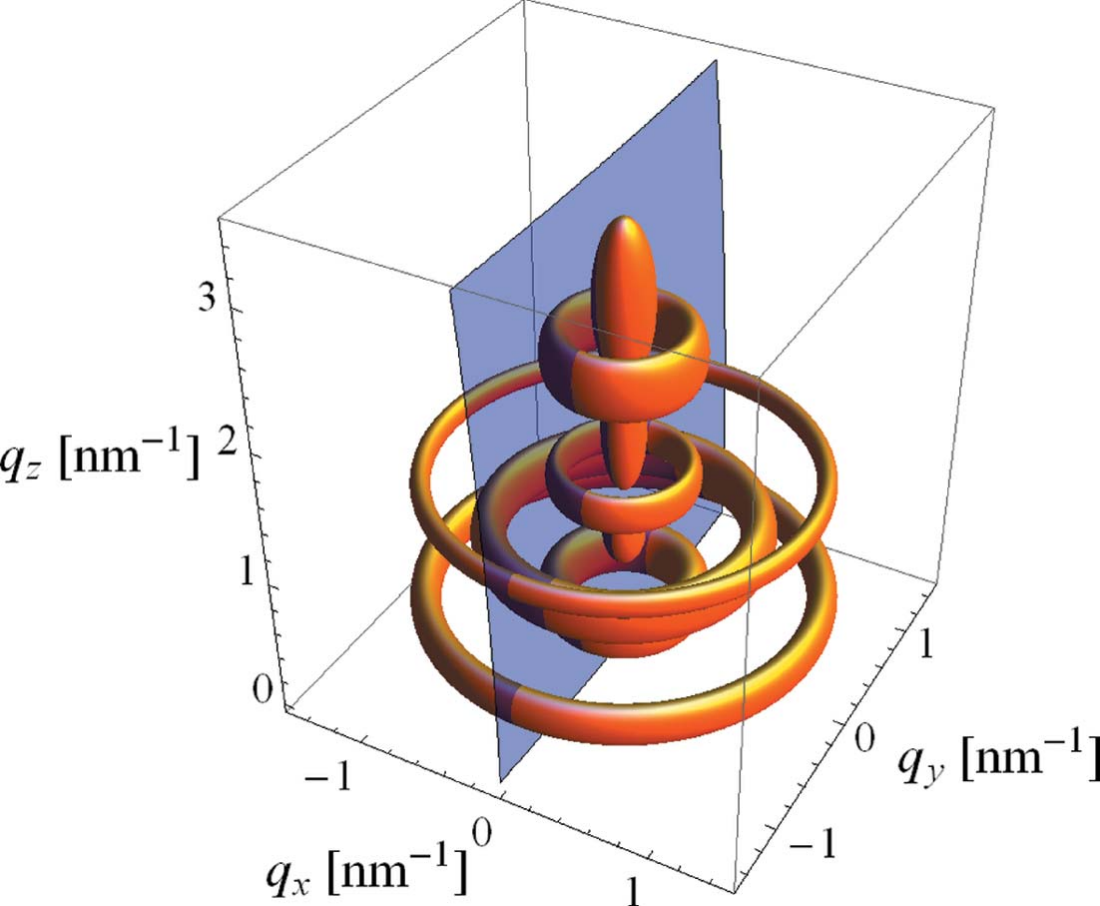
Contours of identical scattering intensity in reciprocal space for the dip-coated sample. The blue surface indicates the Ewald sphere and the cut through reciprocal space measured by the detector.

The total intensity of the peaks was obtained by integration over the peak intensity over the whole reciprocal space by assuming cylindrical symmetry around the *q_z_* axis. Only a cut along the Ewald sphere (Ewald, 1913 ▸) through space at *q_x_* ≃ 0 is seen in GISAXS (Fig. 2 ▸).

**Figure 3:**
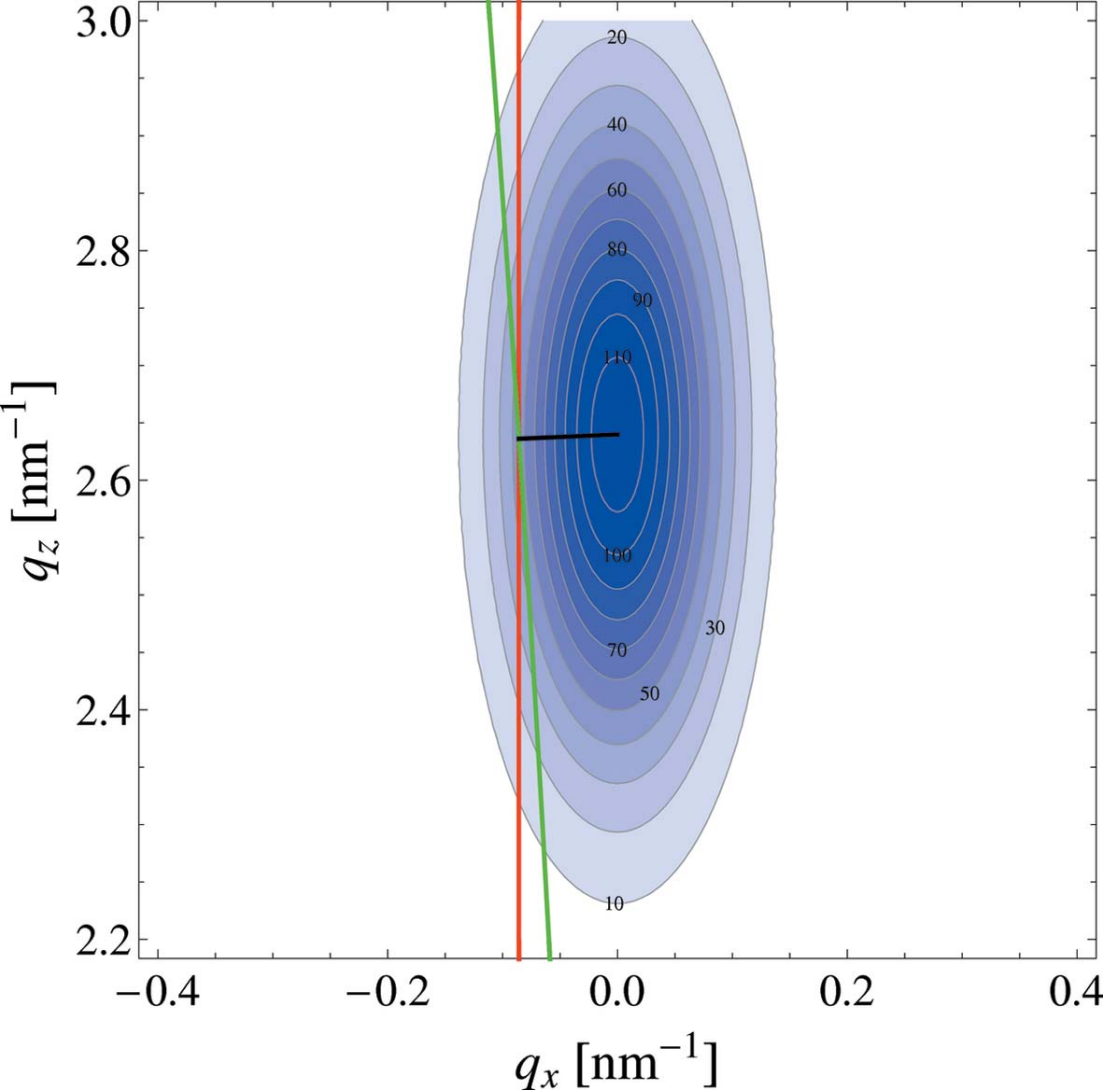
Cut through the (06) peak (dip-coated, 2 mm s^−1^). The green line denotes the Ewald sphere and the red line the plane used to approximate the cut by the Ewald sphere. The black line indicates the distance *q*_*x*,E_. The thickness of the Ewald sphere shell is smaller than the width of the green line.

In most cases, the curvature of the Ewald sphere can be neglected, but one has to correct for the fact that the cut is considerably off the centre of the peak along the (0*k*) line. It is possible to describe the intensity 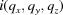 in three-dimensional reciprocal space of a (0*k*) peak centred at 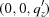 as a three-dimensional Gaussian decay. The lateral distribution within the 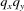 plane is centrosymmetric with a standard deviation *s*_l_, and the vertical standard deviation is *s*_v_. The peak intensity is therefore
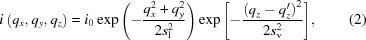
where *i*_0_ is the intensity at the centre of the peak. The Ewald sphere cuts through this distribution and the fitted peak is a two-dimensional approximation to this curved cut. If we neglect the curvature of the Ewald sphere and the tilt of the tangential plane close to the centre of the peak, we approximate the true intersection by that of a plane which intersects the *x* axis at *q*_*x*,E_ and which has a normal vector parallel to the *x* axis (Fig. 3 ▸). This corresponds to
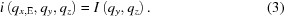
It becomes obvious by setting *q_y_* = 0 and *q_z_* = 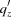 that
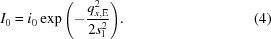
Similarly, setting *q_y_* = σ_l_ reveals that *s*_l_ = σ_l_. Therefore *i*_0_, the value at the centre of the peak, can be obtained as
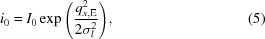
which leads to the correction factor
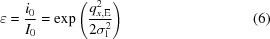
for (0*k*) peaks. For all other peaks ∊ = 1.

The total intensity can be obtained by rotating the image around the *q_z_* axis and integrating:
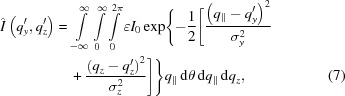
with *q*_||_ = 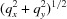.

In the case of peaks (0*k*), the peaks are on the line defined by 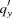 = 0 and the integral results in
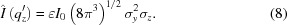
All other peaks correspond to rings in reciprocal space. Their total intensity is therefore 
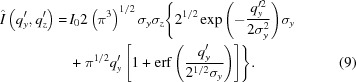
A further correction was applied for the different scattering volumes, depending on the overlap volume of the incoming beam and the observation direction (Smilgies, 2002 ▸).

### 3.2. Determination of pore shape   

Mittelbach & Porod (1961 ▸) have pointed out that the cross-section scattering contribution of an elliptical cylinder in a given direction is identical to that of the affine circle between the two tangents. If we assume that the *a* axis of the ellipse is parallel to the surface of the substrate, then the radius of the affine circle is
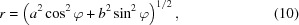
where φ is the angle between the *a* axis and the direction of interest. In our case, φ = 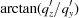. Therefore, we can approximate the experimental data points (*i.e.* the integrated intensities) after some simplification by the expression
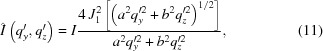
where *J*_1_ is the first-order Bessel function of the first kind.

In practical terms, each peak was approximated ten times by manual selection of a region of the data set where the model of equation (1) was approximated and the results averaged. In all cases except for the (0*k*) peaks, the results of the 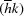 and (*hk*) peaks were averaged.

The limits of uncertainty of the parameters were estimated by a Monte Carlo approach repeating the approximation 100 times, where the intensity values of the points had been shifted. These shifts were random, resulting in a normal distribution with a standard deviation similar to that determined from the experimental data (Svergun & Pedersen, 1994 ▸).

### 3.3. Pore filling from the shift of the Yoneda peak   

The position of the Yoneda peak was determined from a vertical cut of the detector at about *q_y_* = 0.7 nm^−1^, averaging the lateral intensities over a range of ten pixels. The resulting curves showed up to three maxima in the angular range 0.15 < α_f_ < 0.2°, where α_f_ is the vertical component of the scattering angle. The average position was then computed as the centre of mass of this cut.

In the absence of absorption, the refractive index *n* of X-rays is
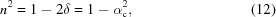
where α_c_ is the critical angle of total reflection and the decrement δ = λ^2^ *r*_e_ρ_e_/(2π) is related to the wavelength λ, the classical electron radius *r*_e_ = 2.81794 × 10^−15^ m and the electron density ρ_e_. This leads to (Forster, 1927 ▸; Prins, 1928 ▸)
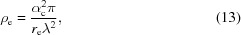
connecting the electron density to the critical angle of total reflection and thereby to the position of the Yoneda peak.

## 4. Results and discussion   

### 4.1. Determination of pore shape   

The GISAXS patterns (Fig. 1 ▸) of the samples show pronounced peaks. The peaks of the dip-coated films prepared with high rates of withdrawal tilt increasingly inward and elongate. This is related to strong worm-like undulations of the cylindrical pores (Grosso *et al.*, 2001 ▸). In the case of the dip-coated film prepared at 10 mm s^−1^, the peaks are already developing towards elliptical rings.

The peaks caused by the beam reflected at the surface of the substrate (Tate *et al.*, 2006 ▸) are weaker but can be observed, *e.g.* the (02) peak of the reflected beam can be seen in the dip-coated film prepared at 10 mm s^−1^ between the ordinary (02) and the (04) peaks [labelled (02)′ in Fig. 1 ▸(*b*)]. The split of the peaks (02) and (02)′ is large, owing to the incidence angle being considerably larger than the critical angle. This is also the reason for the low intensity of the peak (02)′. Therefore, those terms including a reflection event in the DWBA can be neglected and only refraction effects at the surface need to be taken into account.

The systematic absence of all peaks except for *h* + *k* = 2*n* (*n* is an integer) indicates a centrosymmetric unit cell with identical electron densities at positions (*y*, *z*) and (*y* + *A*/2, *z* + *B*/2), where *A* and *B* are the side lengths of the rectangular unit cell in the lateral and vertical directions, respectively. This agrees well with the case of the comparable bulk material SBA-15 (Zhao *et al.*, 2013 ▸), where the hexagonal arrangement in plane group *p*6*mm* can be obtained by a centrosymmetric lattice with *B* = *A*(3^1/2^). However, the peak positions observed in the GISAXS pattern correspond to a different axis ratio where *B*/*A* is considerably smaller than 3^1/2^ (*cf.* Table 1 ▸). This can be explained by shrinkage during film preparation, which is stronger in the vertical than in the lateral direction. This leads to a distortion of the unit cell and a different plane group, namely *c*2*mm* (Crepaldi *et al.*, 2003 ▸).

A casual visual inspection of the scattering pattern reveals that the (13) peaks are systematically weaker than the (15) and (11) peaks. Neither of them is suppressed by the lattice symmetry. The integration over the whole intensity ring in reciprocal space should also lead to comparable effects in total intensity, since their 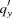, σ_*y*_ and σ_*z*_ values are of comparable magnitude, leading to similar multiplication factors of the observed intensities. Therefore, the form factor of the individual nanostructure must lead to interferences, resulting in a pattern of this kind. This means that one should be able to obtain information on the pore size and shape from the peak intensities.

**Figure 4:**
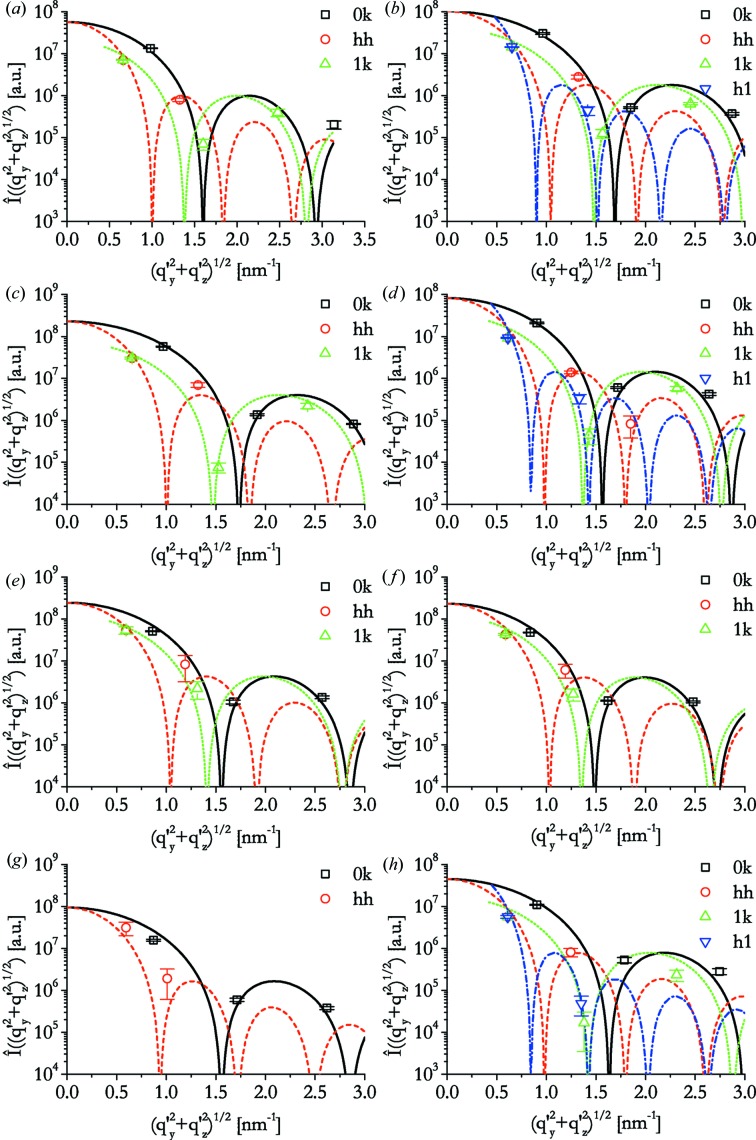
Intensities of peaks including error bars and approximations of the model of elliptical cross section [solid lines: cut through peaks (0*k*); dashed lines: cut through (*hh*); dotted lines: cut through (1*k*); dash–dotted lines: cut through (*h*1)]. (*a*)–(*c*) Spin-coated samples prepared at (*a*) 5000 r min^−1^, (*b*) 2000 r min^−1^ and (*c*) 500 r min^−1^. (*d*)–(*h*) Dip-coated samples prepared at withdrawal speeds of (*d*) 2 mm s^−1^, (*e*) 6 mm s^−1^, (*f*) 8 mm s^−1^, (*g*) 10 mm s^−1^ at low humidity and (*h*) 2 mm s^−1^ at high humidity.

More details can be seen when the total intensities of the peaks are computed according to equations (8) and (9) (points in Fig. 4 ▸). It is evident from these graphs that the intensity of the (11) peak is lower than that of the (02) peak, while the absolute value of the scattering vector is larger for the (02) peak. Assuming homogeneous electron densities within the pores and within the silica matrix, one expects monotonic decay of the forward scattering intensity up to the first minimum of the form factor. Therefore, the observation mentioned indicates that this decay is not identical for all directions of the scattering vector and that the intensity decays less fast in the vertical direction than in directions deviating by about 45°. This suggests that the cross section of the pores deviates from circular, and that the pores are smaller in the vertical direction than in the lateral direction.

The approximation of the elliptical cross-section model resulted in good approximations to the data (see lines in Fig. 4 ▸). The length obtained for the *b* half-axis in the vertical direction is considerably shorter than the *a* axis in the lateral direction for all films investigated (see Table 1 ▸).

The quality of the approximated scattering curves depends mainly on the number of peaks that are available for evaluation. In the best cases (spin coated at 2000 r min^−1^ and dip coated at 2 mm s^−1^), these comprise up to nine reflections [(02), (04), (06), (11), (22), (33), (13), (15) and (31)] and the corresponding symmetric 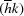. Peaks (31) and (33) were too weak to be determined in all other samples. In some cases, not even the intensities of the strong peaks could be determined in a reliable way, since they overlapped with blind areas of the detector [*e.g.* (04) of the spin-coated film prepared at 5000 r min^−1^] or with rings of the silver behenate standard [*e.g.* (15) for dip-coated samples withdrawn at 6 and 8 mm s^−1^].

In the case of the dip-coated sample at 10 mm s^−1^, only the three peaks in (0*k*) directions could be determined well. Peaks (11) and (22) are the only two that allow for the estimation of the elliptical shape of the pores. They are, however, rather elongated and their total intensity is difficult to determine. Therefore, the results for this sample should be interpreted with caution.

**Figure 5:**
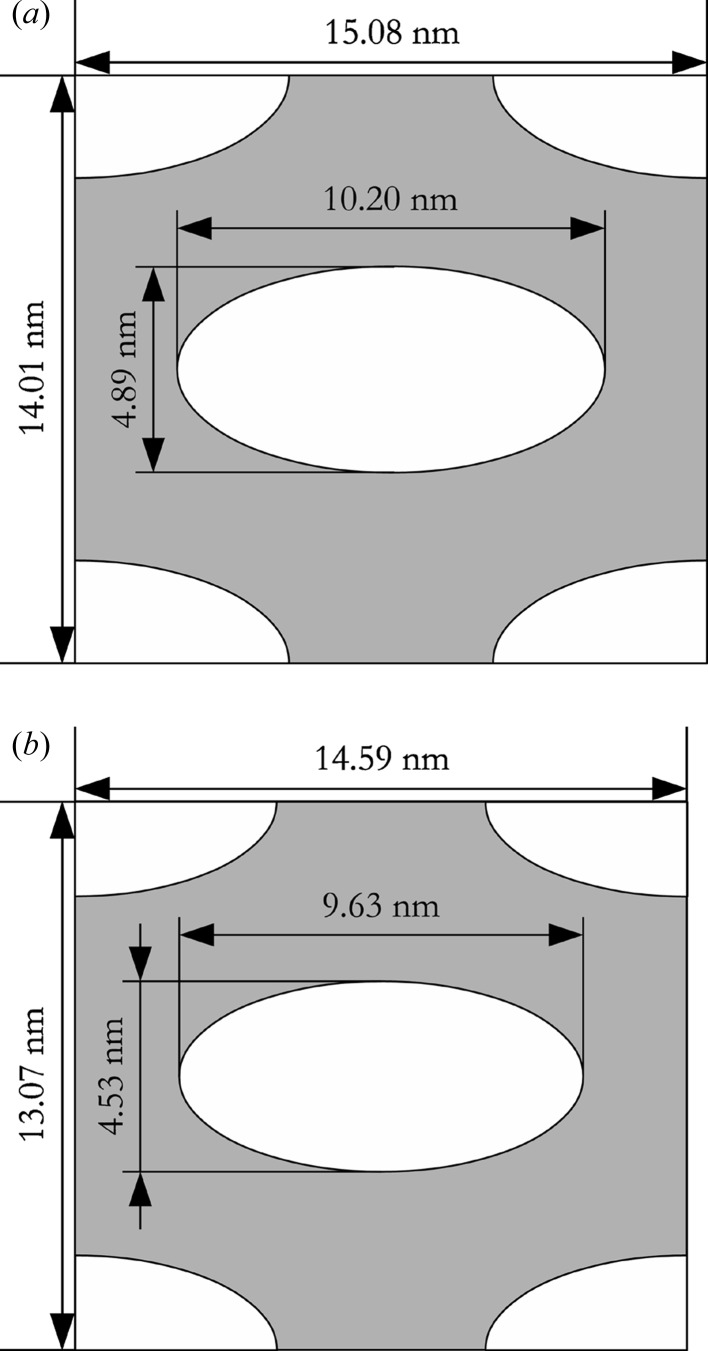
Sketch of unit cells and contained pores. (*a*) Dip-coated sample, withdrawal speed 2 mm s^−1^, (*b*) spin-coated sample, 2000 r min^−1^.

If one computes the deformation of the circular cross section of the original micelles to the elliptical one of the pores as *b*/*a*, one obtains 0.47 for a typical spin-coated film (2000 r min^−1^) and 0.48 for a typical dip-coated film (2 mm s^−1^). This compares well with the deformation one obtains by the deviation of the hexagonal lattice due to shrinkage, as can be seen when relating the lattice constants *A* and *B*. For the same two films, one obtains *B*/(3^1/2^ *A*) = 0.52 for the spin-coated film and *B*/(3^1/2^ *A*) = 0.54 for the dip-coated film (models for the two unit cells are shown in Fig. 5 ▸). The volume fraction of the pores within the film can be computed as 2*ab*π/(*AB*) and is between 0.32 and 0.39 for the various films studied.

If one compares the results for all the films prepared, one sees some weak trends in film structure. The lateral size of the unit cell varies only sightly and no systematic change is obvious, indicating the stabilization of the structure by the substrate. However, the vertical size of the cell increases with decreasing rotation speed for spin-coated films and with increasing withdrawal speed for dip-coated ones. The only exception to this rule is the dip-coated film prepared at 10 mm s^−1^, which is by contrast rather disordered, showing pronounced elliptical rings next to the broad lattice peaks (Fig. 1 ▸ *b*). The half-axes of the pore cross sections show no clear trend with preparation speed, which is also reflected by a lack of trend in volume fraction. However, the vertical *b* half-axis is found to be larger in the dip-coated films than in the spin-coated ones, which might be attributed to the stronger shear forces during spin coating.

### 4.2. Pore filling determined from the Yoneda peak   

If the pores are filled with water in the vapour phase by changing the relative humidity, the scattering contrast between the pores and the silica matrix changes. One test for the stability of the model presented in §4.1 is the comparison of the same film measured at low and high humidity. The dip-coated film (2 mm s^−1^) shows similar peak positions and ratios in dry (Fig. 4 ▸ *d*) and wet (Fig. 4 ▸ *h*) states. It shows slight shrinkage when filled with water, but the axis ratios of the unit cell and of the pore remain basically unchanged (Table 2 ▸).

**Figure 6:**
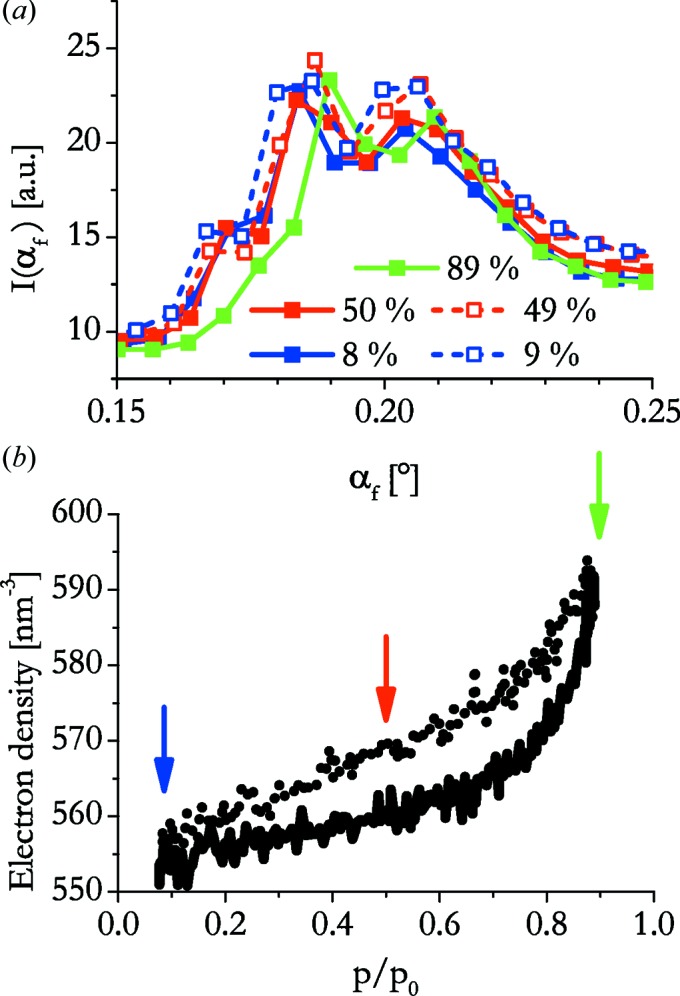
(*a*) The Yoneda peak of the dip-coated film prepared at 6 mm s^−1^ for different relative humidities. Solid lines and filled symbols are used for adsorption, dashed lines and open symbols for desorption. (*b*) The electron density of the film computed from the peak position. The arrows mark the approximate humidities of the curves given in part (*a*)

A vertical cut through the scattering pattern at small angles (*cf.* Fig. 1 ▸) shows a broad peak that is often split into two side maxima at angles between 0.15 and 0.2°, which is demonstrated for the dip-coated sample with a withdrawal speed of 6 mm s^−1^ (Fig. 6 ▸ *a*). The centre of this peak corresponds to an electron density of about 550 e^−^ nm^−3^ for the dry sample and it shifts in humid air to a density of about 590 e^−^ nm^−3^ (Fig. 6 ▸ *b*). Decreasing the humidity shifts the position of the peak to lower angles again, eventually reaching the initial electron density of the film.

If we assume a density of 2.196 g ml^−1^ for the silica after calcination (Haynes, 2011 ▸), we obtain an electron density of 660 e^−^ nm^−3^ for amorphous silica. Taking into account the cross section of the elliptical pores and the unit-cell dimensions (Table 1 ▸), the volume fraction of the silica phase is φ_s_ = 0.68. Multiplying the electron density of the pure bulk material by this value, we get an estimate for the electron density of the porous film, which is 445 e^−^ nm^−3^. The pores are here assumed to be empty. However, if all the pores are completely filled with water, we can estimate the electron density of this layer by adding (1 − φ_s_)ρ_e,water_. The electron density of the water within the pores should not be too far off that of bulk water (Wagner & Pruß, 2002 ▸; Erko *et al.*, 2012 ▸), which was taken as ρ_e,water_ = 334 e^−^ nm^−3^. Therefore, the electron density of the completely filled film should be 554 e^−^ nm^−3^. This difference in electron density is expected to correspond to a shift in position of the Yoneda peak from 0.177 to 0.198°.

The electron densities obtained from the position of the Yoneda peak are somewhat higher than those calculated from the bulk material properties and unit-cell dimensions. This could be caused by a non-homogeneous film, where parts exist that show a different morphology from the highly ordered arrangement of parallel elliptical cylindrical pores. The split of the Yoneda peak and the presence of contributions at angles larger than 0.198° indicate that this is the case.

The electron density as a function of relative vapour pressure corresponds to a sorption isotherm. This is important, since only a few methods allow the nondestructive determination of sorption isotherms of thin films on a substrate [*e.g.* ellipsometric porosimetry (Boissiere *et al.*, 2005 ▸) and X-ray reflectometry (Dourdain & Gibaud, 2005 ▸)].

The sorption isotherm relating the electron density of the pores to the humidity (Fig. 6 ▸) shows a rapid increase at relative humidities larger than approximately 0.75, which becomes rather steep above humidities of 0.88. This is attributed to capillary condensation in the pores. Upon desorption a hysteresis is seen, but the shape of the curve is not quite as expected for this type of mesoporous material. In particular, a sharp drop due to capillary evaporation would be expected. We suspect that the discrepancy is due to experimental problems of not reaching equilibrium during desorption.

## 5. Conclusions   

Films templated by pluronic P123 show shrinkage during calcination. This deforms the unit cell of the originally hexagonally ordered micelles in the vertical direction, leading to a centrosymmetric rectangular unit cell.

The peak intensities from GISAXS data, which correspond to cuts by the Ewald sphere through reciprocal space, can be multiplied by a shape factor, taking into account the cylindrical symmetry of the scattering pattern in three-dimensional space. The resulting points are not influenced by the arrangement of the scattering objects and can be used to estimate the form factor of the pore cross section. The pores are found to be deformed to elliptical cross sections. The amount of deformation is similar to that of the unit-cell axes.

Dip-coated films are generally slightly less deformed than spin-coated ones.

Water can be adsorbed and desorbed by the pores. The amount of water within the film can be monitored by the shift in the critical angle for total reflection during the sorption cycle.

## Figures and Tables

**Table 1:** Film thickness (*t*) and cell parameters for differently prepared films

Film type	Speed	*t* (nm)	*A* (nm)	*B* (nm)	*a* (nm)	*b* (nm)	*B*/[*A*(3^1/2^)]	*b*/*a*
Spin coated	5000 r min^−1^	59 ± 2	14.64	12.72	5.051 ± 0.050	2.395 ± 0.020	0.50	0.474 ± 0.007
	2000 r min^−1^	233 ± 8	14.59	13.07	4.815 ± 0.041	2.266 ± 0.005	0.52	0.471 ± 0.004
	500 r min^−1^	231 ± 10	14.05	13.00	5.019 ± 0.044	2.212 ± 0.003	0.53	0.441 ± 0.004
Dip coated	2 mm s^−1^	70 ± 1	15.08	14.01	5.102 ± 0.049	2.447 ± 0.006	0.54	0.480 ± 0.005
	6 mm s^−1^	71 ± 5	14.93	14.64	4.539 ± 0.164	2.460 ± 0.008	0.57	0.542 ± 0.020
	8 mm s^−1^	64 ± 4	14.73	15.23	4.529 ± 0.120	2.576 ± 0.004	0.60	0.569 ± 0.015
	10 mm s^−1^	77 ± 3	14.78	14.54	5.135 ± 0.383	2.458 ± 0.009	0.57	0.479 ± 0.036

**Table 2:** Cell parameters for a dry and a wet dip-coated film (2 mm s^−1^)

Relative humidity (%)	0.089	0.913
*A* (nm)	15.08	14.98
*B* (nm)	14.01	13.90
*a* (nm)	5.102 ± 0.049	5.125 ± 0.048
*b* (nm)	2.447 ± 0.006	2.346 ± 0.011
*B*/[*A*(3^1/2^)]	0.54	0.54
*b*/*a*	0.480 ± 0.005	0.458 ± 0.005
